# Update Immunglobulin-A-Vaskulitis

**DOI:** 10.1007/s00393-022-01162-z

**Published:** 2022-03-18

**Authors:** Thomas Neumann

**Affiliations:** 1 Klinik für Rheumatologie, Kantonsspital St. Gallen, Rorschacher Str. 95, 9007 St. Gallen, Schweiz; 2 Universität Zürich, Rämistrasse 71, 8006 Zürich, Schweiz

**Keywords:** Purpura Schönlein-Henoch, Kleingefäßvaskulitis, Therapie, Prognose, Manifestationen, Henoch-Schoenlein purpura, Small vessel vasculitis, Treatment, Prognosis, Manifestations

## Abstract

Die Immunglobulin-A-Vaskulitis (IgAV) ist eine systemische Vaskulitis der kleinen Gefäße mit Ig(Immunglobulin)A-Immunkomplexbildung und einem breiten Spektrum klinischer Konstellationen. Typische Manifestationen sind Purpura, Arthralgien oder Arthritiden, Enteritis und Glomerulonephritis. Die IgAV ist die häufigste Vaskulitis im Kindesalter mit meist unkompliziertem und selbstlimitierendem Verlauf. Erwachsene erkranken deutlich seltener an einer IgAV, wobei die Verläufe insbesondere bei renaler oder gastrointestinaler Manifestation komplizierter sind. Verschiedene Trigger der IgAV, darunter Infektionen, wurden beschrieben, wobei eine gestörte Glykosylierung von IgA1 mit konsekutiver Freilegung von Bindungsstellen für Autoantikörper die pathophysiologische Voraussetzung für die Vaskulitis ist. Therapeutische Strategien mit Immunsuppressiva sind bisher mit geringer Evidenz unterlegt, berücksichtigen die Schwere der Organmanifestationen und orientieren sich an den Empfehlungen zur Behandlung anderer Vaskulitiden der kleinen Gefäße. Benigne Verläufe werden symptomatisch behandelt. Die langfristige Prognose der IgAV ist von der renalen Manifestation beeinflusst.

## Abstract (en)

Immunoglobulin A vasculitis (IgAV) is a systemic vasculitis of the small vessels with formation of IgA immune complexes and a broad spectrum of clinical constellations. Typical manifestations include purpura, arthralgia or arthritis, enteritis and glomerulonephritis. The IgAV is the most common vasculitis in childhood with a mostly uncomplicated and self-limiting course. In adults IgAV is much less frequent but the course can be more complicated, especially with renal or gastrointestinal manifestations. Various triggers of IgAV including infections have been described, whereby impaired glycosylation of IgA1 with subsequent exposure of binding sites for autoantibodies is a pathophysiological precondition for the vasculitis. Therapeutic strategies with immunosuppressants are so far supported by low evidence, take the severity of the organ manifestations into account and are oriented towards the recommendations for the treatment of other vasculitides of small vessels. Benign courses are treated symptomatically. The long-term prognosis of IgAV is determined by the renal manifestation.

Die Immunglobulin-A-Vaskulitis (IgAV), früher als Purpura Schönlein-Henoch beschrieben, ist eine leukozytoklastische, immunkomplexvermittelte Vaskulitis der kleinen Gefäße mit charakteristischen Ig(Immunglobulin)A1-dominaten Immunablagerungen. Haut, Gelenke, Gastrointestinaltrakt und Nieren können neben weiteren sehr seltenen Manifestationen (alveoläre Hämorrhagie, Myokarditis, Uveitis, Keratitis, Skleritis, zerebrale Vaskulitis, periphere Neuropathie, Orchitis) betroffen sein. Die IgAV ist mit einer jährlichen Inzidenz von 3–26,7 pro 100.000 die häufigste Vaskulitis im Kindesalter [11, 41]. Das mittlere Alter bei Erstmanifestation liegt bei 6 Jahren. Für Erwachsene wird eine jährliche Inzidenz von 0,8–1,8 pro 100.000 angenommen [41, 58]. Das mittlere Manifestationsalter liegt bei 50 Jahren. Epidemiologische Daten sind aufgrund der Heterogenität der angewendeten Klassifikationskriterien nur eingeschränkt vergleichbar. Die IgAV verläuft meist selbstlimitierend und bedarf lediglich einer symptomorientierten Therapie. Allerdings müssen organ- oder lebensbedrohende Komplikationen aggressiv immunsuppressiv behandelt werden.

Die erste Beschreibung eines 4 Jahre alten Jungen mit Arthritis, Purpura, gastrointestinaler und renaler Beteiligung erfolgte 1802 durch William Heberden. Im Jahr 1837 erkannte Johann Schönlein den Zusammenhang zwischen Purpura und Arthralgien, und Eduard Henoch ergänzte 1874 die Beteiligung des Gastrointestinaltraktes und der Nieren [17, 49].

Die renale Manifestation der IgAV und die sehr viel häufigere IgA-Nephropathie (IgAN) weisen histologische Gemeinsamkeiten auf. Auch die IgAN ist durch glomeruläre IgA-Ablagerungen und mesangial proliferative Veränderungen charakterisiert, allerdings fehlen systemische Manifestationen. Es resultiert die Hypothese, dass IgAN und IgAV Varianten derselben Erkrankung sind. Unterstützt wird diese Vermutung durch familiäre Cluster mit IgAN und IgAV [7]. Während sich jedoch die Nephritis im Rahmen einer IgAV als akutes nephritisch/nephrotisches Syndrom präsentiert, verläuft die IgAN meist schleichend und kann auch als zufälliger histologischer Befund bei der Abklärung einer asymptomatischen Hämaturie auffallen. Patienten mit renaler IgAV sind jünger als bei der IgAN und haben meist eine raschere Verschlechterung der Nierenfunktion [50].

## Diagnose und Klassifikation

Die Diagnose der IgA wird nach klinischen und histologischen Kriterien gestellt. Weitere Vaskulitiden der kleinen Gefäße müssen differenzialdiagnostisch abgegrenzt werden. Obwohl bei bis zu 50 % der Patienten ein erhöhtes IgA im Serum vorliegt und bei einigen Patienten eine Komplementverminderung besteht, sind diese Laborparameter als diagnostisches Kriterium zu unspezifisch [48].

In der ACR(American College of Rheumatology)-Klassifikation von 1990 wurden erstmals Kriterien für die IgAV aufgestellt (Tab. 1; [31]). Diese Kriterien basieren auf der Auswertung einer Kohorte von 807 Patienten mit Vaskulitis, darunter 85 mit IgAV. In einer gemeinsamen Initiative der European League against Rheumatism (EULAR), der Paediatric Rheumatology International Trials Organization (PRINTO) und der Paediatric Rheumatology European Society (PRES) wurden 2010, basierend auf Daten von 827 Kindern, neue Klassifikationskriterien für die IgAV bei Kindern vorgeschlagen [37]. Im Vergleich zu den ACR-Kriterien (1990) wurde das Kriterium des Patientenalters herausgenommen, und die prädominanten IgA-Ablagerungen, die Nieren- und Gelenkbeteiligung wurden hinzugefügt. Dadurch wird eine Sensitivität von 100 % erreicht. Obwohl diese Kriterien für die Diagnose der IgAV im Kindesalter entwickelt wurden, finden sie auch bei Erwachsenen aufgrund des Fehlens spezifisch validierter Kriterien Anwendung. In einer Kohorte von 129 Erwachsenen mit IgAV stellte sich unter Anwendung der EULAR/PRINTO/PRES-Kriterien eine Sensitivität von 99,2 % und eine Spezifität von 86 % dar, die damit höher lag als die der ACR-Kriterien [18].

**Table 1:** 

ACR-Klassifikationskriterien (1990)	EULAR/PRINTO/PRES-Klassifikationskriterien 2008	Chapel Hill Consensus Conference (CHCC) 2012
Alter ≤ 20 Jahre bei Krankheitsbeginn	Palpable Purpura, dominierend an den unteren Extremitäten	Vaskulitis mit IgA1-dominanten Ablagerungen
Palpable Purpura	Diffuse, kolikartige abdominelle Schmerzen mit akutem Beginn (Invagination oder Blutung möglich)	Betroffen sind die kleinen Gefäße (Kapillaren, Venolen oder Arteriolen) der Haut und des Gastrointestinaltraktes
Akute abdominelle Schmerzen	Leukozytoklastische Vaskulitis mit dominierend IgA-Ablagerungen oder proliferative Glomerulonephritis mit IgA-Ablagerungen	Arthritiden sind häufig
Histologischer Nachweis von Granulozyten in der Wand der kleinen Arteriolen oder Venolen	Arthritis mit akutem Beginn (Schmerzen, Schwellung und Funktionseinschränkung), Arthralgien mit akutem Beginn (Schmerzen ohne Schwellung oder Funktionseinschränkung)	Glomerulonephritis (nicht von der IgA-Nephropathie zu unterscheiden) kann auftreten
–	Proteinurie > 0,3 g/24 h oder > 30 mmol/mg Albumin/Kreatinin-Ratio im Spontanurin, Hämaturie oder Erythrozytenzylinder: > 5 Erythrozyten/HPF oder Erythrozytenzylinder oder Proteinurie ≥ 2+ im Spontanurin	–
≥ 2 von 4 Kriterien	Kriterium 1 (obligatorisch) und ≥ 1 weiteres Kriterium	–
Sensitivität 87,1 %, Spezifität 87,7 %	Sensitivität 100 %, Spezifität 87 %	–

*HPF* „High Power Field“, Hauptgesichtsfeld

Die internationale Chapel Hill Consensus Conference (CHCC) definierte erstmals 1994 und in einer Überarbeitung 2012 die IgAV, wobei 2012 auf das Eponym „Purpura Schönlein-Henoch“ zugunsten der IgAV verzichtet wurde [23].

## Pathogenese

Die genaue Pathophysiologie der IgAV ist nicht aufgeklärt. Die derzeitige Hypothese geht von einer in genetisch prädisponierten Individuen durch Umweltfaktoren getriggerten und durch ein verändertes IgA fehlgeleiteten Immunantwort aus. Unterstützt wird diese Annahme durch die saisonal unterschiedliche Häufigkeit des Auftretens der Erkrankung und meist vorausgehende Infektionen. Fraglich ist bisher, ob eine genetische Prädisposition die spezifische Ausprägung der Erkrankung und den Verlauf beeinflusst. Die Pathophysiologie der renalen Manifestation der IgAV weist wesentliche Gemeinsamkeiten mit der IgAN auf [50]. Charakteristisch sind die Bildung von IgA1-Immunkomplexen und die Ablagerung in den kleinen Gefäßen, insbesondere in der Haut, der Niere und dem Gastrointestinaltrakt. In der sog. Hinge-Region von IgA1 finden sich 3 bis 6 Bindungsstellen, an denen in einer O‑Glykosylierung Galaktose über N‑Acetylgalactosamine an Serin oder Threonin gebunden wird. Varianten von kritischen Enzymen führen zu einer verminderten Glykosylierung und der Bildung von Galaktose-defizientem IgA1 (Gd-IgA1). Autoantikörper erkennen Galaktose-defiziente O‑Glykane, binden an diese als Neoepitope erkannte Strukturen und bilden darüber Immunkomplexe mit Gd-IgA1. Die Bildung der Immunkomplexe ist der kritische Schritt in der Pathogenese [59]. Gd-IgA1 lässt sich in der Niere und der Haut bei der IgAV nachweisen, und erhöhte Gd-IgA1-Serumspiegel sind mit der Krankheitsaktivität und mit dem Auftreten einer renalen Beteiligung assoziiert [33, 53, 62]. Eine Aktivierung der Komplementkaskade ist an der Gewebeschädigung beteiligt. Erhöhte C3c- und C5a-Fragmente und Ablagerungen von C3 und C5–9 weisen auf eine Aktivierung der alternativen Komplementkaskade hin [60]. Das für die IgAN postulierte Modell von 4 „Hits“ ist auch auf die renale Manifestation der IgAV übertragbar. Infolge der Bildung von Gd-IgA1, Entstehung Gd-IgA1-spezifischer IgG-Autoantikörper, Immunkomplexbildung und Aktivierung mesangialer Zellen mit konsekutiver Freisetzung inflammatorischer Mediatoren entwickelt sich der glomeruläre Schaden [16].

Unterschiedliche Inzidenzen in verschiedenen ethnischen Gruppen legen eine Bedeutung genetischer Faktoren in der Pathogenese der IgAV nahe [41]. Zwei Genom-weite Assoziationsstudien (GWAS) zeigen Veränderungen in den HLA-Klasse-II-Genen gegenüber Gesunden [26, 27]. López-Mejías et al. wiesen an 285 spanischen Patienten mit der IgAV im Vergleich zu 1006 Kontrollen einen Polymorphismus in der Region zwischen HLA-DQA1 und DQB1 nach [27]. Es konnte auch gezeigt werden, dass sich der genetische Hintergrund zwischen IgAV und IgAN nicht wesentlich unterscheidet. Mehrere weitere Polymorphismen, die nicht in *HLA*-Genen liegen, darunter IL(Interleukin)-1-Rezeptor-Antagonist Allel 2 und IL‑8, wurden beschrieben [2, 3]. IL‑8 ist bedeutsam in der Rekrutierung von Neutrophilen.

## Klinische Assoziationen

Die IgAV kann assoziiert mit Infektionen, malignen Erkrankungen, Autoimmunerkrankungen oder mit der Einnahme von Medikamenten auftreten. Bei Kindern lassen sich eine jahreszeitliche Häufung der Manifestation im Herbst und Winter sowie in den meisten Fällen eine vorausgehende Infektion im oberen und unteren Respirationstrakt und im Gastrointestinaltrakt nachweisen [10]. Im Gegensatz dazu ist diese Assoziation bei Erwachsenen seltener zu beobachten [10]. Häufige Pathogene sind Staphylokokken, Streptokokken, *Helicobacter* (*H.*) *pylori*, Parvovirus und Hepatitis-B-Virus. Es sind mehrere Fälle einer IgAV infolge einer COVID-19-Infektion beschrieben [52]. Die IgAV wurde auch nach Impfungen beobachtet, darunter aktuell nach einer COVID-19-Impfung [36]. In Pharmakovigilanzdaten findet sich neben Impfungen eine Assoziation mit Antibiotika (β-Lactam-Antibiotika, Fluorchinolone und Makrolide) und TNF(Tumor-Nekrose-Faktor)-Blockern [45]. Ein weiterer Zusammenhang ist mit malignen Erkrankungen beschrieben [40, 63]. In den meisten Fällen liegen solide Malignome vor. Die Patienten mit Tumor-assoziierter IgAV sind älter, haben häufiger eine nekrotisierende Purpura, eine Hämaturie, alveoläre Hämorrhagie und konstitutionelle Symptome [15]. Möglicherweise trägt die Assoziation mit malignen Erkrankungen zu der in 2 Studien beobachteten Übersterblichkeit für Patienten mit einer IgAV im Vergleich zur Allgemeinbevölkerung bei [40, 57]. Darüber hinaus sind Assoziationen einer IgAV mit entzündlichen Darmerkrankungen, Spondyloarthritiden und familiärem Mittelmeerfieber beschrieben [1, 38].

## Manifestationen

Die Erstmanifestation der IgAV im Kindes- oder Erwachsenenalter bedingt meist einen spezifischen Verlauf. Während abdominelle Schmerzen und Arthritis häufiger bei Kindern auftreten, ist eine kutane Vaskulitis bei Kindern und Erwachsenen meist vorhanden. Für die renale Beteiligung gibt es keine dominierende Altersgruppe, allerdings ist der Verlauf im Erwachsenenalter meist ungünstiger [28, 57]. Die Erkrankung manifestiert sich innerhalb von Tagen oder Wochen in unterschiedlicher Abfolge an den betroffenen Organen oder tritt als sog. „single organ disease“ z. B. an der Niere oder Haut auf.

### Haut

**Figure 1:**
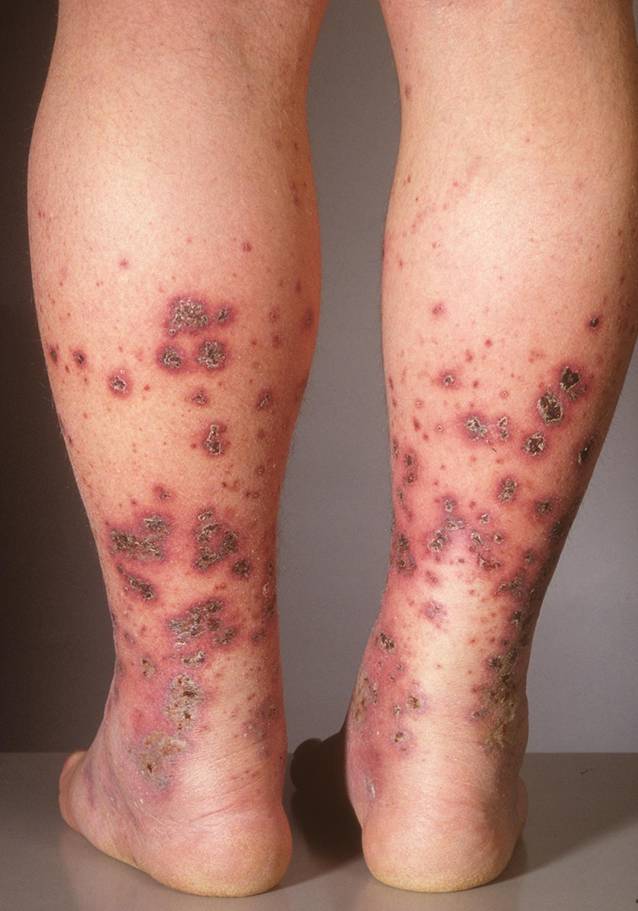

Eine kutane Manifestation wird im Initialstadium bei 75 % und im gesamten Verlauf der Erkrankung bei 100 % der Patienten beschrieben und präsentiert sich als Purpura mit lokalisierten subkutanen Ödemen (Abb. 1; [4, 5]). Bei einem Drittel der Erwachsenen kommt es zu einer nekrotisierenden oder hämorrhagischen Purpura. Die kutane Vaskulitis kann in Schüben verlaufen und dauert ungefähr 3 Wochen. Während die Hautläsionen bei Kindern meist an den unteren Extremitäten auftreten, sind bei Erwachsenen häufig der Körperstamm und die unteren Extremitäten betroffen [25].

### Gelenke

Arthralgien und Arthritiden sind bei ca. 84 % der Kinder und Erwachsenen mit IgAV vorhanden [5]. Es imponieren Weichteilschwellungen und schmerzhafte Gelenke, während eine Arthritis seltener auftritt. Typisch sind passagere, oligoartikuläre, nicht destruierende Verläufe, die häufiger die großen Gelenke der unteren Extremitäten betreffen. Auch Myalgien werden beschrieben.

### Gastrointestinaltrakt

Bei der Hälfte der Kinder und Erwachsenen kommt es in einem variablen Intervall nach der kutanen Manifestation zu einer gastrointestinalen Beteiligung [4, 25]. Die Symptome variieren von milden (abdominale Schmerzen, Übelkeit, Erbrechen) bis zu schweren Verläufen (Hämorrhagien, Ischämien, Nekrosen, Invagination, Perforation). Invaginationen sind insbesondere bei Kindern bekannt [12].

### Niere

Renale Manifestationen mit Proteinurie, Hämaturie und Erythrozytenzylindern sind bei Erwachsenen (76,2 %) häufiger und treten insbesondere in Kombination mit gastrointestinalen Manifestationen und nekrotisierender kutaner Vaskulitis auf [61]. Bei bis zu 30 % der Erwachsenen kann initial eine eingeschränkte Nierenfunktion bestehen, während dies nur bei weniger als 1 % der Kinder beobachtet wird [40, 61].

**Figure 2:**
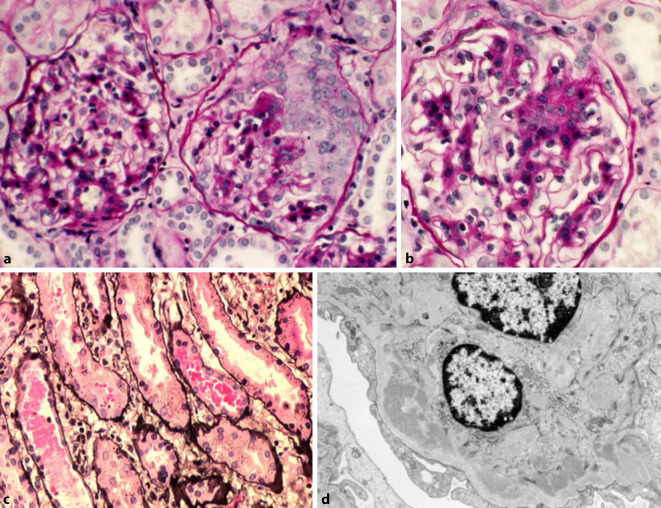

Eine Nierenbiopsie ist zur Sicherung der Diagnose und zur Beurteilung der renalen Schädigung von zentraler Bedeutung (Abb. 2). Der histologische Befund an der Niere orientiert sich an der Klassifikation von Pillebout et al. mit Einteilung in 5 histologische Klassen, die eine klinische Korrelation zeigen [40]. Ungünstige renale Prognosefaktoren bei Baseline sind: eingeschränkte glomeruläre Filtrationsrate (GFR), Proteinurie > 1 g/Tag, makroskopische Hämaturie, arterielle Hypertonie und eine im Verlauf persistierende Proteinurie > 1 g/Tag [40, 51]. Diese klinischen Parameter korrelieren mit dem Vorhandensein einer interstitiellen Fibrose und sklerotischen Läsionen in der Biopsie. Während die renale IgAV bei Kindern meist ausheilt, treten rezidivierende Verläufe im Erwachsenenalter in etwa 20 % auf [4, 56]. Rezidive der IgAV wurden auch nach Transplantationen mit einer Häufigkeit von 11,5–60 % beobachtet, was in 0–50 % zu einem Verlust der Transplantatniere führt [14, 24].

### Weitere Manifestationen

Selten tritt eine pulmonale Beteiligung mit alveolärer Hämorrhagie auf. Die Prävalenz wird mit 0,8–5 %, allerdings mit einer Mortalität von 28 %, angegeben [32]. Eine Myokarditis, Arrhythmien oder Valvulitis wurden in seltenen Fällen beschrieben [42]. Weitere mögliche Manifestationen sind Orchitis, Uveitis, Keratitis, Skleritis, zerebrale Vaskulitis oder periphere Neuropathie [61].

## Therapiestrategien

**Figure 3:**
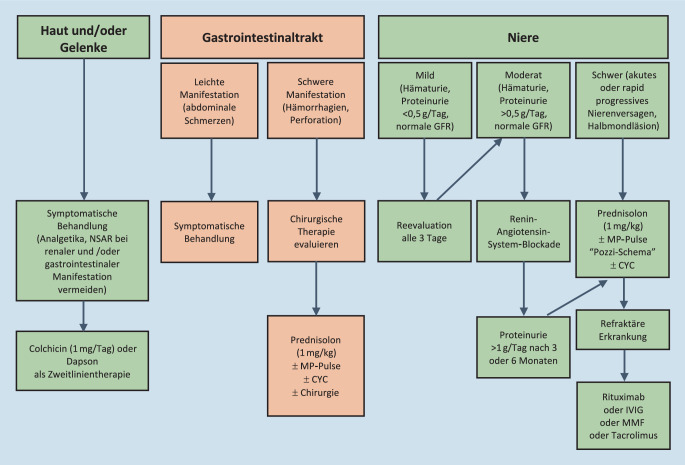

Das therapeutische Management der IgAV orientiert sich am klinischen Phänotyp und berücksichtigt den meist benignen und selbstlimitierenden Verlauf der Erkrankung (Abb. 3). Eine nicht nekrotisierende Purpura und Arthralgien werden symptomatisch analgetisch behandelt. Dabei sollten nichtsteroidale Antiphlogistika (NSAR) aufgrund der potenziellen gastrointestinalen und renalen Beteiligung der Erkrankung vermieden werden. Günstiger sind Paracetamol oder Metamizol. Schwere Verläufe, insbesondere bei gastrointestinaler und renaler Beteiligung, werden mit Kortikosteroiden allein oder in Kombination mit weiteren immunsuppressiven Medikamenten behandelt. Es besteht jedoch nur eine geringe Evidenz für die immunsuppressive Therapie, und diese basiert v. a. auf pädiatrischen Studien. Auch für die Dauer der Therapie existiert keine evidenzbasierte Empfehlung. Für die IgAN konnte in der STOP-IgAN-Studie kein zusätzlicher Effekt einer immunsuppressiven Therapie gegenüber einer bestmöglichen unterstützenden Behandlung dargestellt werden [46].

ACE(„angiotensin converting enzyme“)-Hemmer sollten bei milder bis moderater Proteinurie und arterieller Hypertonie immer eingesetzt werden.

### Colchicin

Liegt eine isolierte kutane Manifestation vor, dann kann Colchicin (Startdosis 2‑mal 0,5 mg/Tag, ggf. steigern auf 3‑mal 0,5 mg/Tag) eingesetzt werden [47]. In einer derzeit laufenden kontrollierten Studie (COLCHIVAS, NCT04008316) wird der Effekt einer 6‑monatigen Therapie mit 1 mg Colchicin mit einem Beobachtungszeitraum von 12 Monaten untersucht.

### Dapson

Eine Alternative für Patienten mit isolierter kutaner IgAV ist Dapson (Dosis 1–2 mg/kg Körpergewicht oder 50–150 mg/Tag). Eine Glukose-6-phosphat-Defizienz sollte zuvor geprüft werden. In einer laufenden kontrollierten klinischen Studie (ARAMIS, NCT02939573) wird derzeit die Therapie mit Dapson, Colchicin oder Azathioprin bei IgAV und anderen ausschließlich kutanen Vaskulitiden untersucht [30].

### Kortikosteroide

Der sinnvolle Einsatz von Kortikosteroiden bei der IgAV wird aufgrund der geringen Evidenz und der potenziellen Nebenwirkungen kontrovers diskutiert. Bei Patienten mit nicht nekrotisierender Purpura, Arthralgien und milder renaler Beteiligung (mikroskopische Hämaturie, Proteinurie < 1 g/Tag, normale Nierenfunktion) sind Kortikosteroide nicht empfohlen. Es existieren lediglich pädiatrische Daten, die bezüglich renaler Rezidive und gastrointestinalen Outcomes widersprüchliche Ergebnisse zeigten [8, 21, 22]. Da es keine Daten aus kontrollierten klinischen Studien für Erwachsene mit IgAV gibt, orientiert sich das Management der renalen Manifestation an der Behandlung der IgAN. Die Kidney Disease: Improving Global Outcomes (KDIGO) empfiehlt für Patienten mit moderater und schwerer Proteinurie und einer GFR > 50 ml/min das Pozzi-Schema (1 g Methylprednisolon an 3 konsekutiven Tagen jeweils der Monate 1, 3 und 5 und orales Prednisolon 0,5 mg/kg an alternierenden Tagen für 6 Monate) [43, 44].

### Azathioprin

Es liegen keine Daten für die Therapie mit Azathioprin (AZA) bei Erwachsenen mit IgAV vor, jedoch zeigen Daten aus kleinen unkontrollierten Studien bei pädiatrischen Patienten, dass sich der klinische Verlauf der Nephritis unter AZA verbessert [9]. Darüber hinaus zeigte sich auch ein Ansprechen der leukozytoklastischen Vaskulitis der Haut [9]. AZA kann alternativ zu Dapson oder Colchicin bei ineffektiver symptomatischer Therapie der kutanen Manifestation eingesetzt werden.

### Mycophenolat-Mofetil

Mehrere Studien zeigen einen positiven Effekt von Mycophenolat-Mofetil (MMF) hinsichtlich der Remissionsinduktion bei Patienten mit ANCA(antineutrophile zytoplasmatische Antikörper)-assoziierter Vaskulitis (AAV) oder IgAN [19, 54]. Basierend auf dem Vorliegen ähnlicher pathologischer Veränderungen bei der AAV, IgAN und IgAV wurde MMF in 2 Fallserien bei Kindern mit Kortikosteroid-resistenter Nephritis mit dem Ziel der Remissionsinduktion und der Vermeidung von Rezidiven erfolgreich eingesetzt [34]. Retrospektive Daten zum Effekt von MMF im Vergleich zu einer Monotherapie mit Kortikosteroiden bei Erwachsenen mit IgAV und renaler Beteiligung zeigen ein schnelleres Ansprechen von MMF bei gleicher Gesamtremissionsrate, aber weniger Nebenwirkungen [13].

### Ciclosporin A

Nach einigen Fallberichten wurde Ciclosporin A (CsA) in einer kontrollierten Studie an 24 pädiatrischen Patienten mit schwerer Nierenbeteiligung im Vergleich zu Methylprednisolon-Pulsen, gefolgt von Prednisolon, über 12 Monate untersucht [20]. Dabei zeigten sich unter CsA eine schnellere Remissionsinduktion und eine niedrigere Rezidivrate als unter Prednisolon-Monotherapie. Für erwachsene Patienten liegen nur wenige Fallberichte vor, die keine abschließende Einschätzung zulassen.

### Cyclophosphamid

In Analogie zu den Erfahrungen in der Behandlung anderer Vaskulitiden mit rapid progressiver Glomerulonephritis wird Cyclophosphamid (CYC) auch bei der IgAV bei lebens- oder organbedrohenden Verläufen, insbesondere bei Patienten mit renaler Beteiligung, eingesetzt. Die einzige randomisierte Studie bei Kindern mit IgAV-assoziierter Nephritis zeigte jedoch keinen positiven Effekt hinsichtlich des renalen Outcomes für CYC zusätzlich zu supportiven Maßnahmen [55]. Bei Erwachsenen mit renaler und gastrointestinaler Beteiligung wurde CYC im Vergleich zu hoch dosierten Kortikosteroiden randomisiert und kontrolliert untersucht [39]. Auch in dieser Studie zeigte sich kein Unterschied in den Remissionsraten nach 6 und 12 Monaten. Die Daten sollten jedoch aufgrund der kleinen Fallzahl mit Vorsicht interpretiert werden. In der umfangreichsten retrospektiven Auswertung der französischen Vaskulitisarbeitsgruppe (French Vasculitis Study Group) war ebenfalls kein zusätzlicher Effekt für CYC nach den ersten 12 Monaten zu erkennen [4].

### Rituximab

Die Therapie mit Rituximab (RTX) ist Standard in der Remissionsinduktion und -erhaltung bei der AAV. Basierend auf diesen Erfahrungen wird RTX in refraktären Verläufen der IgAV eingesetzt. Eine monozentrische Auswertung für 22 erwachsene Patienten mit refraktärer IgAV zeigte eine Remissionsrate von 91 % nach 6 Monaten und ein gutes Ansprechen der renalen Manifestation [29].

## Prognose

Die IgAV verläuft meistens unkompliziert und heilt bei Kindern in 94 % und bei Erwachsenen in 89 % der Patienten komplett aus [5, 6]. Besteht allerdings eine Nierenbeteiligung, dann ist die Prognose deutlich ungünstiger, und eine komplette renale Remission (keine Proteinurie oder Hämaturie und normale Nierenfunktion) wird bei Erwachsenen nur in 20 % nach 15 Jahren erreicht [6, 40]. Eine chronische Nierenerkrankung stellt somit eine wesentliche Komplikation der IgAV dar [40]. Rezidive werden in 20 % der erwachsenen Patienten beschrieben, wobei extrarenale Manifestationen im Rezidiv häufiger auftreten (gastrointestinal 16 %, Purpura 41 %, Arthralgien 11 %) [25, 51].

Eine chronische Nierenerkrankung stellt eine wesentliche Komplikation dar

Das Risiko eines langfristig komplizierten Verlaufs der IgAV ist assoziiert mit spezifischen Organmanifestationen, wobei renale, pulmonale oder gastrointestinale Manifestationen bei erwachsenen Patienten auch zu einer höheren Morbidität und Mortalität führen [35]. Die Überlebenswahrscheinlichkeit bei Erwachsenen mit IgAV liegt bei 73 % nach 5 Jahren, 62 % nach 10 Jahren und 45 % nach 20 Jahren und damit signifikant unterhalb der gesunden Bevölkerung (Gesamtmortalität: HR [Hazard Ratio] 2,05; 95 %-CI [Konfidenzintervall]: 1,69–2,49) [35]. Ein höherer Carlson-Komorbiditätsindex zu Baseline (HR 1,88; 95 %-CI: 1,25–2,73), Niereninsuffizienz (HR 1,48; 95 %-CI: 1,04–2,22), schwere Infektionen (HR 1,48; 95 %-CI: 1,01–2,16) und höheres Lebensalter (HR 1,05; 95 %-CI: 1,03–1,06) waren unabhängig mit der Mortalität assoziiert [35].

## Fazit für die Praxis

- Die Immunglobulin-A-Vaskulitis (IgAV) ist eine systemische Vaskulitis der kleinen Gefäße mit Ig(Immunglobulin)A-Immunkomplexbildung und einem breiten Spektrum klinischer Konstellationen.
- Haut, Gelenke, Gastrointestinaltrakt und Nieren können neben weiteren sehr seltenen Manifestationen (alveoläre Hämorrhagie, Myokarditis, Uveitis, Keratitis, Skleritis, zerebrale Vaskulitis, periphere Neuropathie, Orchitis) betroffen sein.
- Typische Manifestationen sind Purpura, Arthralgien oder Arthritiden, Enteritis und Glomerulonephritis.
- Die IgAV verläuft meist selbstlimitierend und bedarf lediglich einer symptomorientierten Therapie. Allerdings müssen organ- oder lebensbedrohende Komplikationen aggressiv immunsuppressiv behandelt werden.
- Die langfristige Prognose der IgAV ist von der renalen Manifestation beeinflusst.
